# Vascular Surgery, Microsurgery and Supramicrosurgery for Treatment of Chronic Diabetic Foot Ulcers to Prevent Amputations

**DOI:** 10.1371/journal.pone.0074704

**Published:** 2013-09-13

**Authors:** Steffen Schirmer, Ralf-Gerhard Ritter, Hisham Fansa

**Affiliations:** 1 Department of Plastic, Reconstructive and Aesthetic Surgery, Handsurgery, Klinikum Bielefeld, Bielefeld, North- Rhine Westphalia, Germany; 2 Department of Vascular and Endovascular Surgery, Klinikum Bielefeld, Bielefeld, North- Rhine Westphalia, Germany; Sapienza, University of Rome, School of Medicine and Psycology, Italy

## Abstract

**Introduction:**

Diabetic foot ulcers occur in approximately 2,5% of patients suffering from diabetes and may lead to major infections and amputation. Such ulcers are responsible for a prolonged period of hospitalization and co- morbidities caused by infected diabetic foot ulcers. Small, superficial ulcers can be treated by special conservative means. However, exposed bones or tendons require surgical intervention in order to prevent osteomyelitis. In many cases reconstructive surgery is necessary, sometimes in combination with revascularization of the foot.

There are studies on non surgical treatment of the diabetic foot ulcer. Most of them include patients, classified Wagner 1-2 without infection. Patients presenting Wagner 3D and 4D however are at a higher risk of amputation. The evolution of microsurgery has extended the possibilities of limb salvage. Perforator based flaps can minimize the donorsite morbidity.

**Patients and Methods:**

41 patients were treated with free tissue transfer for diabetic foot syndrome and chronic defects. 44 microvascular flaps were needed. The average age of patients was 64.3 years. 18 patients needed revascularization. 3 patients needed 2 microvascular flaps. In 6 cases supramicrosurgical technique was used.

**Results:**

There were 2 flap losses leading to amputation. 4 other patients required amputation within 6 months postoperatively due to severe infection or bypass failure. Another 4 patients died within one year after reconstruction. The remaining patients were ambulated.

**Discussion:**

Large defects of the foot can be treated by free microvascular myocutaneous or fasciocutaneous tissue transfer. If however, small defects, exposing bones or tendons, are not eligible for local flaps, small free microvascular flaps can be applied. These flaps cause a very low donor site morbidity. Arterialized venous flaps are another option for defect closure.

Amputation means reduction of quality of life and can lead to an increased mortality postoperatively.

## Introduction

In 1989 the St.-Vincent Declaration set the objective that the amputation rate of patients with diabetes should decline by 50% in 5 years [1]. That goal was never accomplished. The number of patients with diabetes mellitus is rising, considering the fact that there were 65.700 major amputations in the USA in 2011 according to the Center of Disease Control (CDC). Worldwide an anticipated 300 million people will suffer from diabetes by 2025 [2]. Although it is only speculative, but this also implies, that the number of diabetes related amputations are going to rise as well.

Aside from endocrinologists, plastic and vascular surgeons have also become an important part of the team treating the problems associated with diabetes, which cause diabetic foot syndrome. It is very well understood that diabetic neuropathy is the major cause of ulcers. In identifying these patients and treating them with neurolysis has shown to prevent ulcers and therefore amputations [3]. Another cause for ulcers is vascular disease. In almost every diabetic patient, who had undergone a major amputation, a peripheral arterial disease was present [4]. Mal perforans or local wounds can be treated by local wound therapy. Numerous wound dressings are in use. Topical use of autologous platelets has also been described [5]. Majority of clinical studies focus on defects classified as Wagner/Armstrong stadium 1 and 2. However, in some cases local wound therapy is not indicated, especially in infected wounds with exposed bones, tendons, vessels or nerves. These wounds are classified as 3D or 4D (Wagner/Armstrong). In many cases chronic osteomyelitis is present, which complicates treatment. A lot of the affected patients are still treated by amputation. Once a major amputation is performed, it is very likely that the contralateral extremity also requires an amputation or a higher level of amputation has to follow. Additionally, there is a high mortality rate after major lower extremity amputation. Only 39% of patients survive 7 years postoperative [6,7].

Therefore, preventing amputation in patients with diabetic foot syndrome can help to maintain quality of life and reduce amputation associated illnesses. It could also help reduce costs. Although this is still a very controversial issue, which depends on the national health system of each country, it is believed that limb salvage can help reduce costs [8].

A radical surgical débridement is necessary and should be performed quickly. Additionally, an antibiotic therapy has to begin [9]. In many cases a defect remains which has to be covered by a free microvascular tissue transfer. Well vascularized tissue does not only cover the defect but it also helps treating osteomyelitis [10]. The concept of free tissue transfer has become an option for limb salvage in elderly patients suffering from diabetes. The success rate of free microvascular tissue transfer as described in publications on older diabetic patients is promising [11,12].

The problem arises if a free flap cannot be performed with desired success due to vascular limitations of the lower extremity. In these cases plastic surgeons have established a so called A-V-loop. This loop is created from autologous veins (e.g. greater saphenous vein) which are anastomosed proximally to a non compromised artery and a vein, thus creating a loop. The loop is then placed distally and the free flap, which covers the defect, is hooked up to the loop. Using this technique the loop does only perfuse the flap and not the ischemic extremity. Although flap surviving is increased the demands of revascularisation are not respected.

To optimize the outcome for the patient a combined approach is necessary: revascularisation of the ischemic leg by providing bypass surgery, if necessary as distal as to the plantar arteries, and in turn the free flap transfer which is hooked end-to-side to the bypass and a end-to-end to a local vein. Using this technique the coverage becomes more successful by increasing the arterial flow and reducing distal resistance. Commonly used free flaps are muscle flaps such as latissimus dorsi, gracilis or rectus abdominis flaps. Fascio-cutaneous flaps include parascapular and scapular flaps. Recently, perforator flaps have also been used in non-traumatic defect cases, mainly the anterior lateral thigh (ALT) flap. These standard flaps are easy to harvest, but cause a significant donor site morbidity in this multi-morbid patient group. In addition, the standard flaps are difficult to shape and place as they sometimes have to be larger than required due to their vascular pattern. Especially this can interfere with the required footwear.

However, since Koshima introduced the concept of supramicrosurgical free flaps, smaller flaps with smaller arteries and veins offer a new perspective for special defects. He described the successful anastomosis of vessels with a diameter of smaller than 1 mm. These small vessels are able to vascularize a flap with a size of up to 21x20 cm [13]. Besides the small vessels, these flaps usually have a very short pedicle [14]. This offers new possibilities for those patients, who suffer from defects over exposed tendons, bones or nerves, which are not suitable to local flaps. In these flaps donor site morbidity is usually low. Another important indication for a small microvascular flap is the need for a second free flap on the same foot. In most cases one of the major arteries or the bypass vessel has been used for the first flap. In order to maintain perfusion of the foot, other vessels have to be chosen. Smaller flaps, such as a peroneus brevis muscle flap, are vascularized by vessels with a smaller diameter. The peroneus brevis muscle can be used as a pedicled flap or as a free microvascular flap [15,16,17].

A third flap procedure is the arterialized venous flap. This technique represents further possibilities for covering defects. The arterial inflow is realized over an afferent vein and the venous outflow through an efferent vein [18]. The procedure was first published by Nakayama in 1981 [19]. Harvesting these flaps causes very low donor site morbidity, which has made it an attractive option for small defects on the hand. However, it is not limited to the hand as described by Woo [18]. When based on the saphenous vein it can be used as bypass and skin flap in one procedure in the above mentioned criteria of revascularisation and coverage.

This study describes our experience with standard microvascular- and supramicrovascular free flap closure in combination with bypass surgery of wounds caused by diabetic foot syndrome.

## Patients and Methods

This is a retrospective outcome study conducted in Germany. The authors contacted The Research Ethics Committee of the Chamber of Physicians Westfalen- Lippe and the Medical Faculty of the Westfalian Wilhelms University, Münster, Germany. It was concluded, that no approval of the committee was necessary for this retrospective study. Furthermore there was no need for an informed consent by the patients using routine data for scientific purposes according to § 6(2) health data protection act NRW (Gesundheitsdatenschutzgesetz Nordrhein-Westfalen). However, all patients approved in publishing the results of this study in either a written or oral consent. In case of oral consent the approval was documented in the patients file.

Since 2007, 74 patients with a diabetic foot syndrome have been treated. 41 of them required a free microvascular tissue transfer for wound coverage (44 flaps). The average age was 64.3 years (31-85y). 3 patients were treated with 2 flaps. 2 of them needed 2 flaps for the ipsilateral foot, another patient needed an additional free flap for the contralateral foot. 18 patients underwent vascular reconstruction either prior to reconstruction or in the same time as the reconstruction took place. Bypass surgery was performed by the vascular surgeon according to the revascularization needs. The overall- time of surgery was not analysed due to the different sizes and localisations of the defects and vascular situation of the leg.

Defects were located on the heel (16), plantar (25) and on the malleolar region (3). Table 1 illustrates the patients, the localisation of the defect and the reconstructive procedure as well as the vascular reconstruction. Patients were classified ASA 3 according to the American Society of Anesthesiology. 3 patients were suffering from an end-stage renal disease and needed hemodialysis. Another patient already had a major amputation on the contralateral leg. One patient was treated under spinal anaesthesia due to cardiac insufficiency. A variety of microvascular flaps were used: There were 23 parascapular flaps, 3 split thickness skin graft (STSG) covered gracilis muscle flaps, 6 latissimus dorsi muscle flaps with STSG, 5 anterior lateral thigh flaps (ALT). 4 patients were treated with a free peroneus muscle brevis flap (1 of them with a skin island, 3 with a STSG), 1 patient was reconstructed with a contralateral instep flap of the contralateral foot, that required an amputation; another patient was reconstructed with a free arterialized venous flap from the thigh and one patient was treated with a free extensor digitorum brevis muscle flap with STSG. In all cases of bypass reconstruction, flap arteries were anastomosed to the bypass in an end-to-side manner. If no bypass was necessary, large flaps, such as parascapular, latissimus dorsi muscle, gracilis muscle, or ALT were anastomosed to pedal arteries (posterior tibial artery, dorsalis pedis artery), mostly in end-to-side technique.

**Table 1:** Demonstrating patients age and sex, defect localization, vascular and plastic surgery procedures.

Sex	Age	Localization	Bypass	Flap
Male	55	Plantar	No	Parascapular
Male	62	Heel	No	Parascapular
Male	54	Heel	Popliteo-Pedal	Arterialized Venous Flap
Male	75	Heel	No	Peroneus brevis
Female	68	Heel	Popliteo- Malleolar	Parascapular
Male*	60	Plantar	Popliteo- Pedal	Parascpular
Male*	61	Plantar	No	Parascapular
Male	69	Heel	No	Parascapular
Male	68	Plantar	No	Parascapular
Female	62	Plantar	FemoroCrural	Latissimus dorsi
Male	64	Heel	No	Parascapular
Male	64	Heel	No	ALT
Male	53	Plantar	No	ALT
Male	74	Plantar	No	Gracilis
Male	74	Heel	Popliteo- Malleolar	ALT
Female	69	Heel	Popliteo- Crural	Parascapular
Female	66	Plantar	Femoro- Popliteal	ALT
Male	55	Plantar	No	Latissimus dorsi
Male**	65	Plantar	No	Parascapular
Male**	65	Malleolus lateralis	No	Peroneus brevis
Female	31	Plantar	No	Peroneus brevis
Male	71	Heel	Femoro- Pedal	Parascapular
Male	74	Heel	Femoro- Crural	Parascapular
Male	67	Heel	No	Latissimus dorsi
Male	70	Plantar	No	Peroneus brevis
Female	58	Plantar	No	Contralateral Instep
Male***	57	Heel	No	Parascapular
Male***	60	Plantar	No	Latissimus dorsi
Male	66	Plantar	Popliteo- Pedal	ALT
Male	71	Malleolus lateralis	Femoro- Pedal	Parascapular
Male	72	Heel	Popliteo- Pedal	Parascapular
Male	32	Heel	Femoro- Popliteal	Latissimus dorsi
Male	69	Plantar	Femoro- Popliteal	Parascapular
Female	63	Plantar	No	Parascapular
Male	51	Plantar	No	Parascapular
Male	70	Plantar	Femoro- Pedal	Parascapular
Male	78	Plantar	Femoro- Pedal	Parascapular
Female	80	Malleolus lateralis	No	Gracilis
Female	59	Heel	No	Parascapular
Male	71	Plantar	Popliteo- Pedal	Parascapular
Male	74	Plantar	Popliteo- Pedal	Parascapular
Male	85	Plantar	AV- Loop	Gracilis
Male	69	Plantar	No	Latissimus dorsi
Female	49	Plantar	No	Extensor digitorum brevis

* patient with an additional flap on the contralateral foot

** patient with an additional flap on the ipsilateral foot

*** patient with an additional flap on the ipsilateral foot

**Figure 1:**
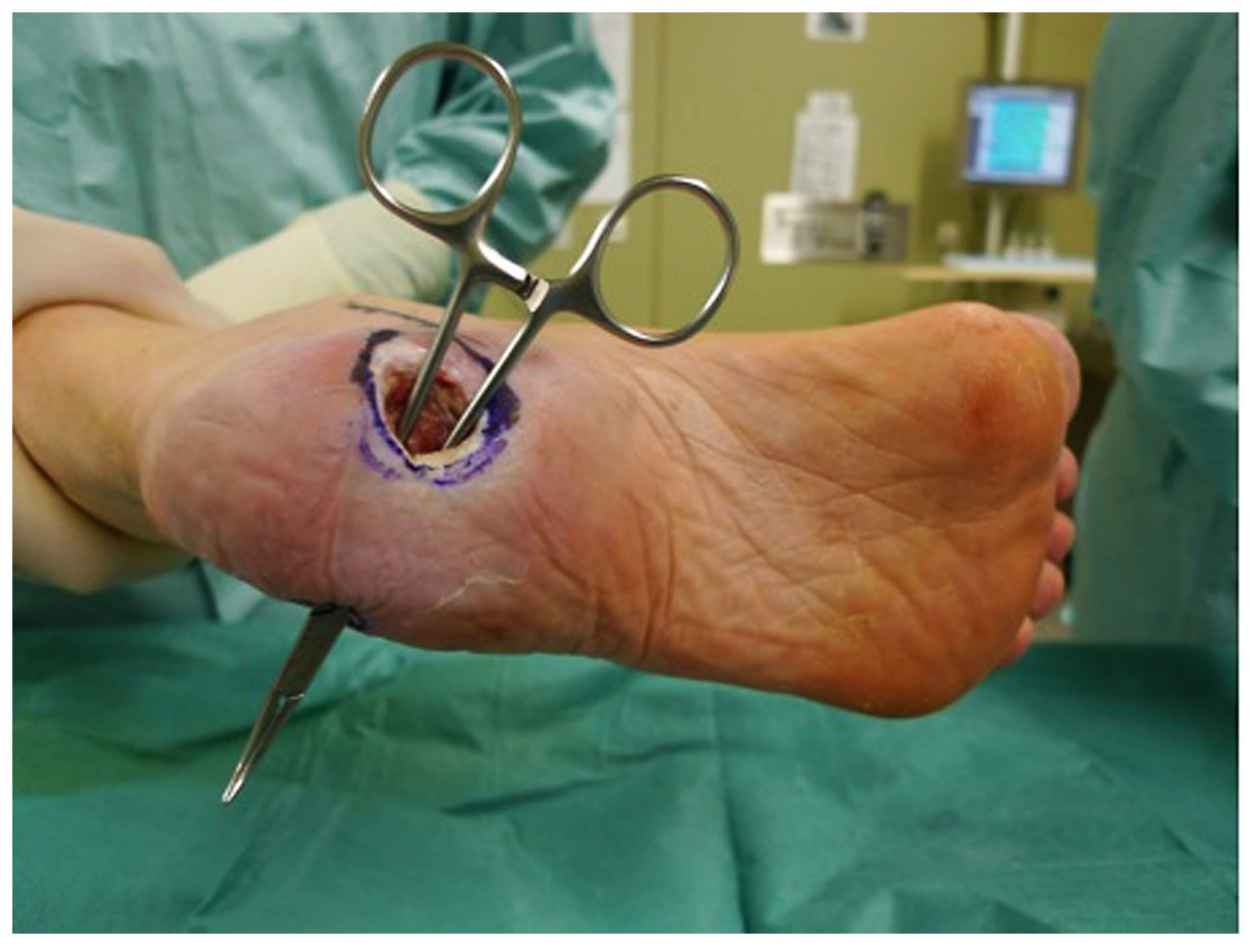
55y.old patient with diabetes and peripheral arterial disease and defect of the heel and exposed bones. The patient required flap and bypass. A reversed greater saphenous bypass was planned including an arterialized venous flap. The bypass supplies the flap by arterial means. 2 separate veins are anastomosed to provide venous outflow.

**Figure 2:**
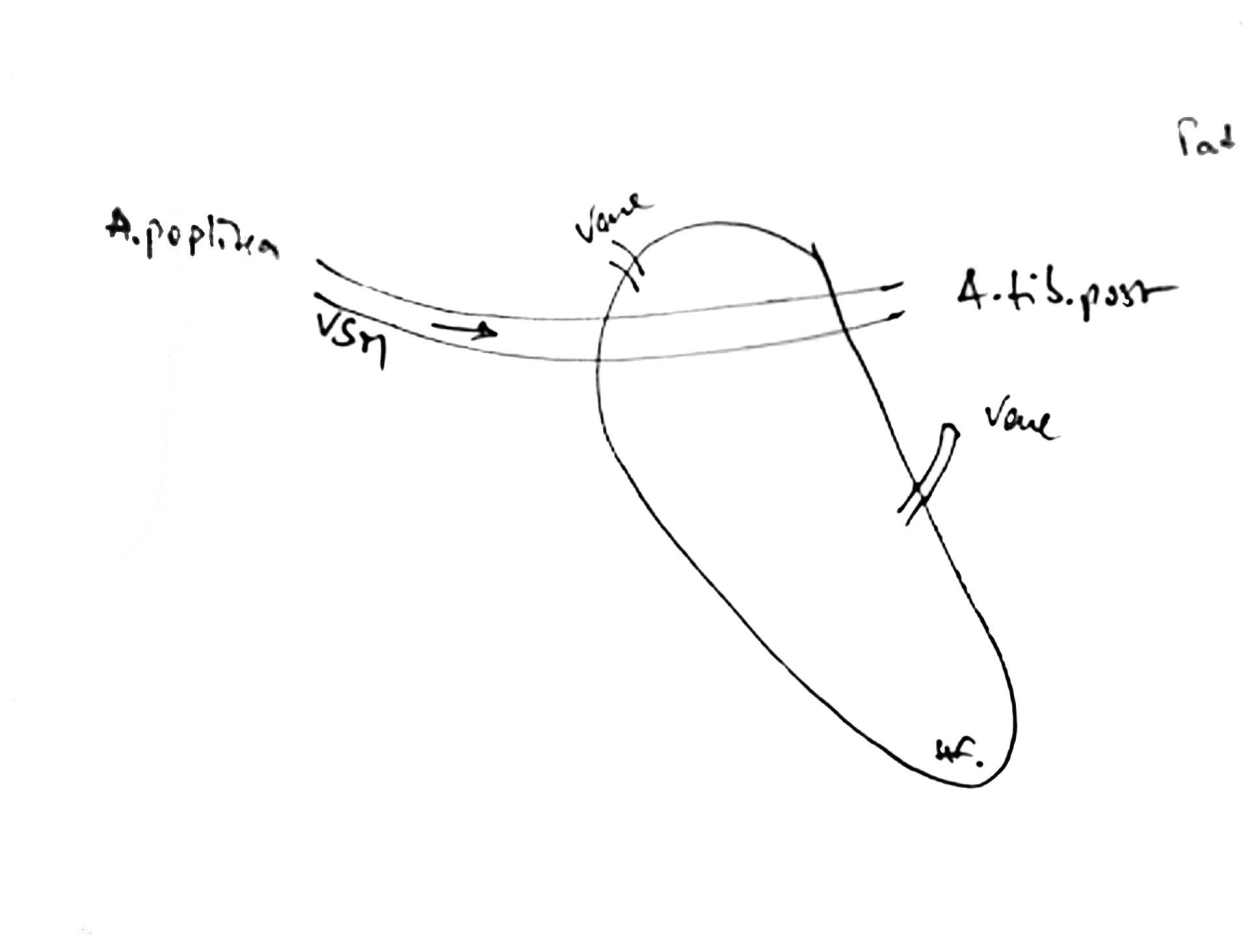
Preoperative planning of the arterialized venous flap. Bypass is anastomosed to the popliteal artery proximally, and the medial plantar artery distally.

**Figure 3:**
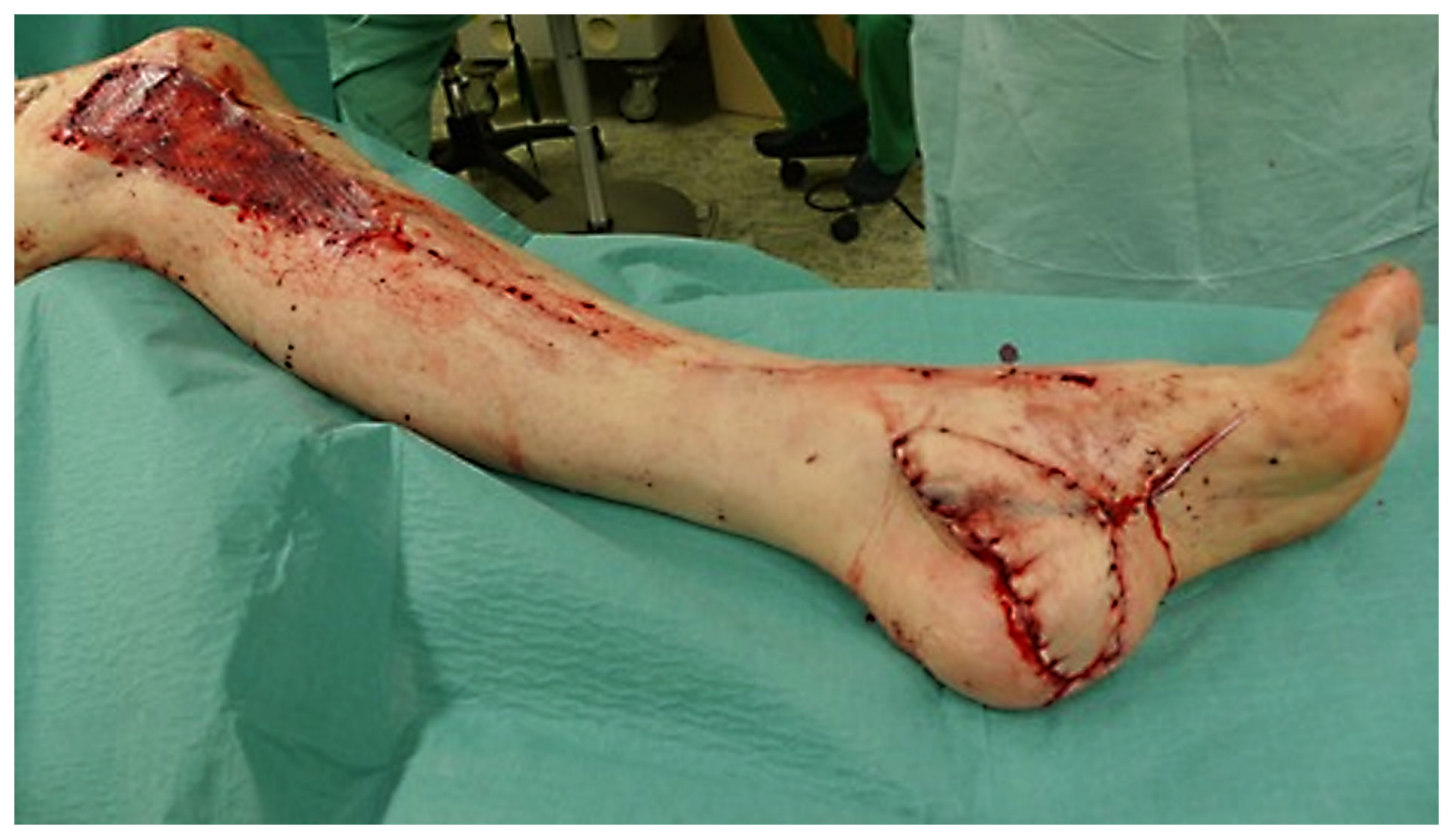
Intraoperative situs: the donor site proximally is covered with a skin graft. The flap covers the distal defect.

**Figure 4:**
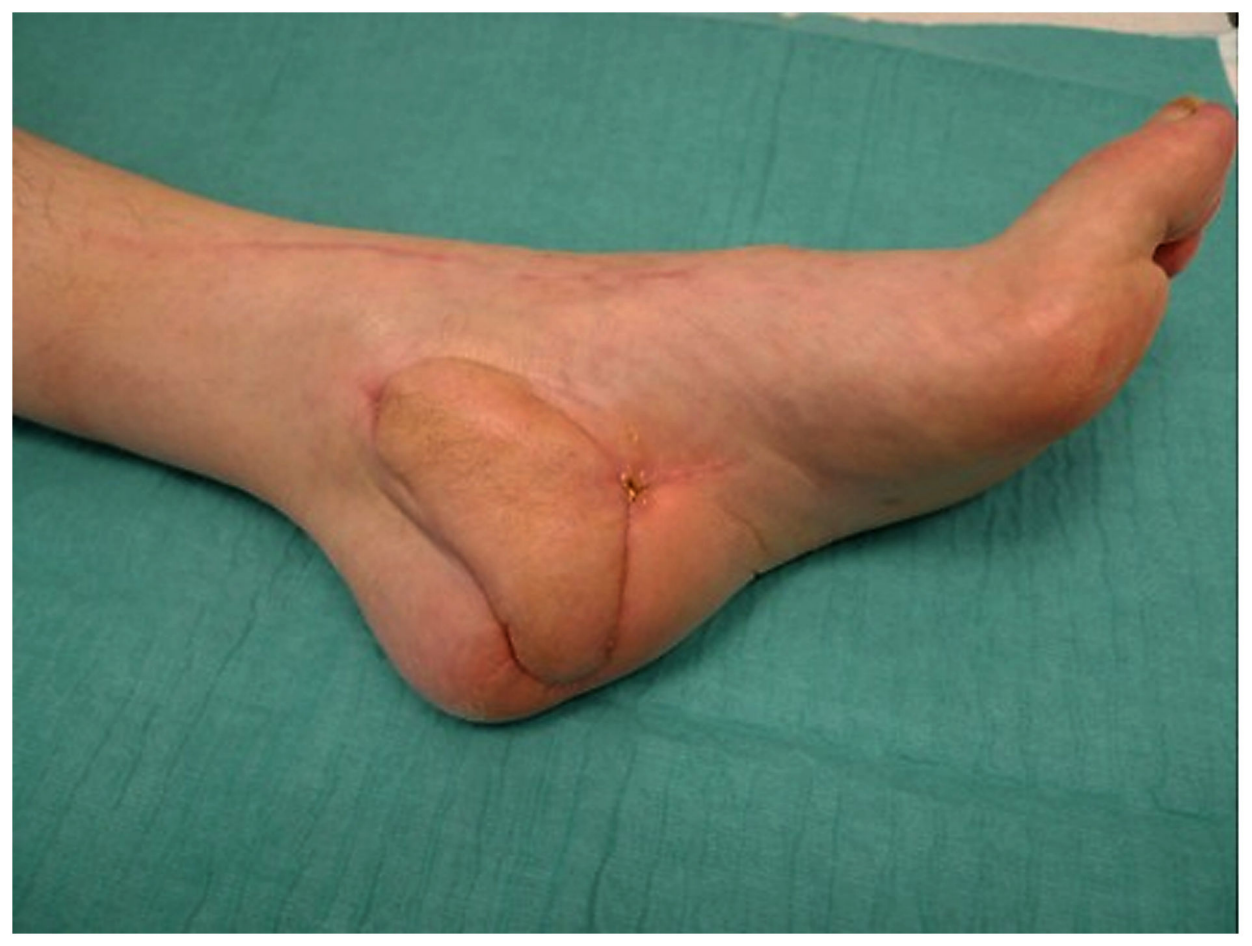
Arterialized venous flap 6 months postoperative.

**Figure 5:**
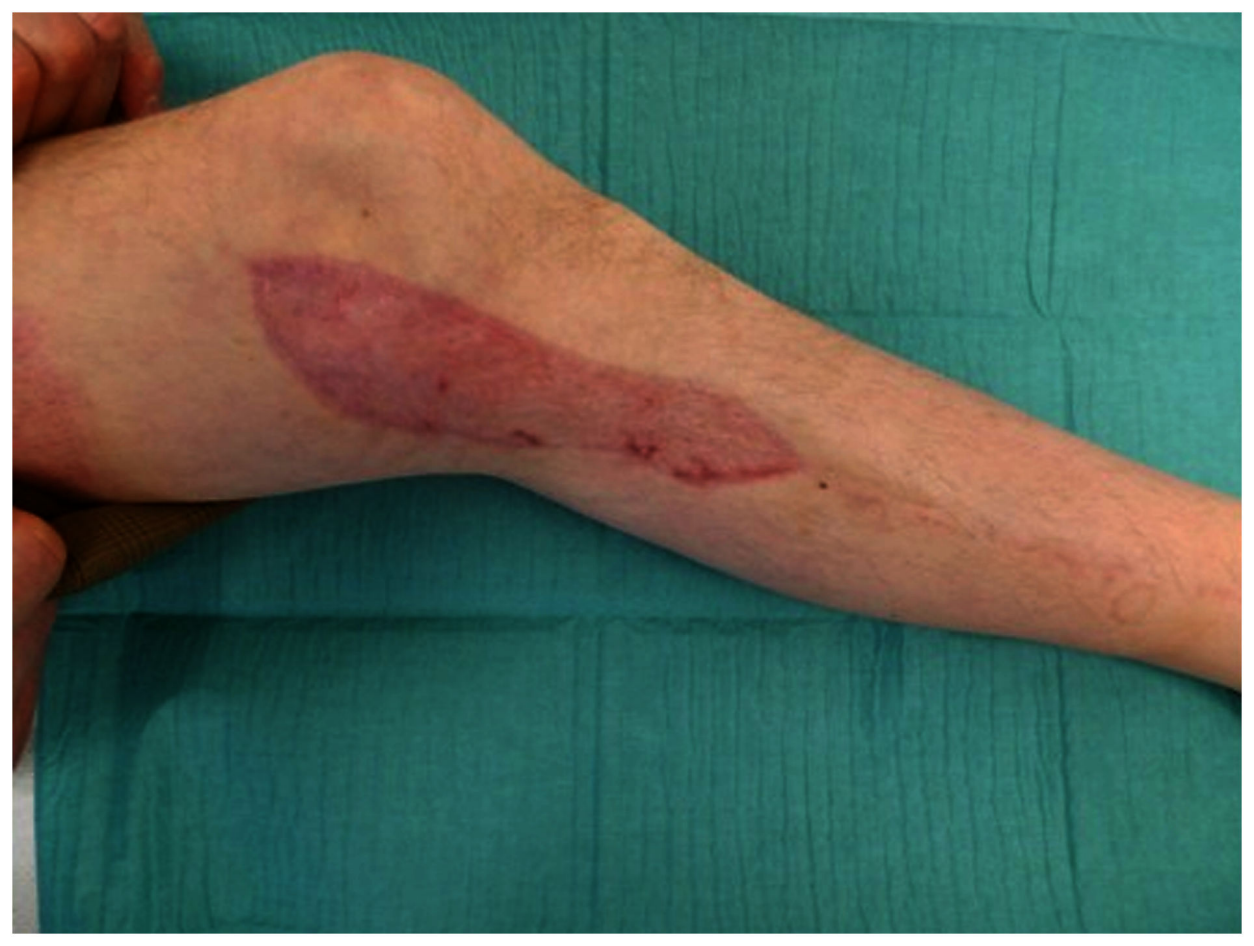
Donor site of the arterialized flap 6 months postoperative.

**Figure 6:**
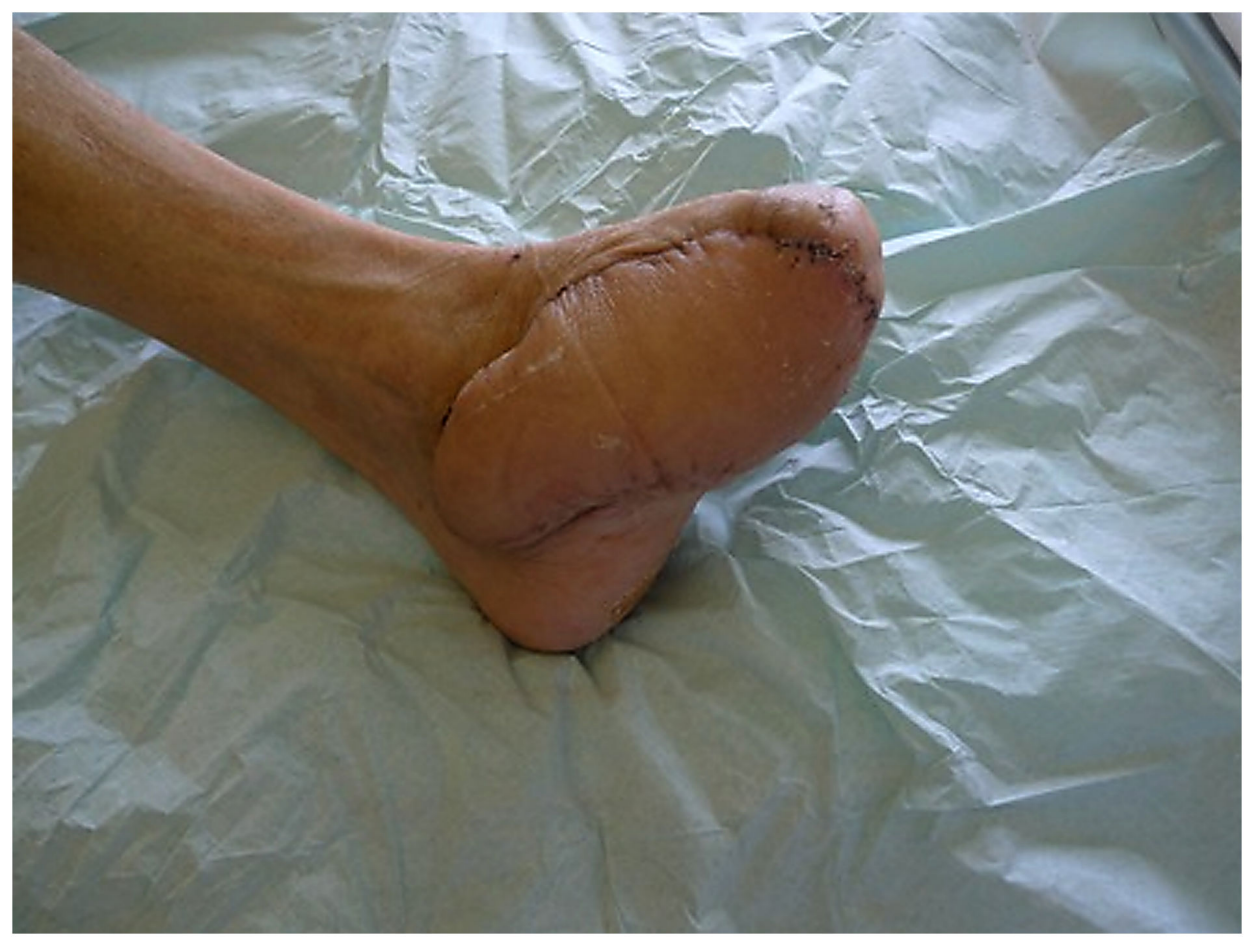
60 y. old patient with diabetes after reconstruction with a parascapular flap covering exposed bone and plantar surface.

**Figure 7:**
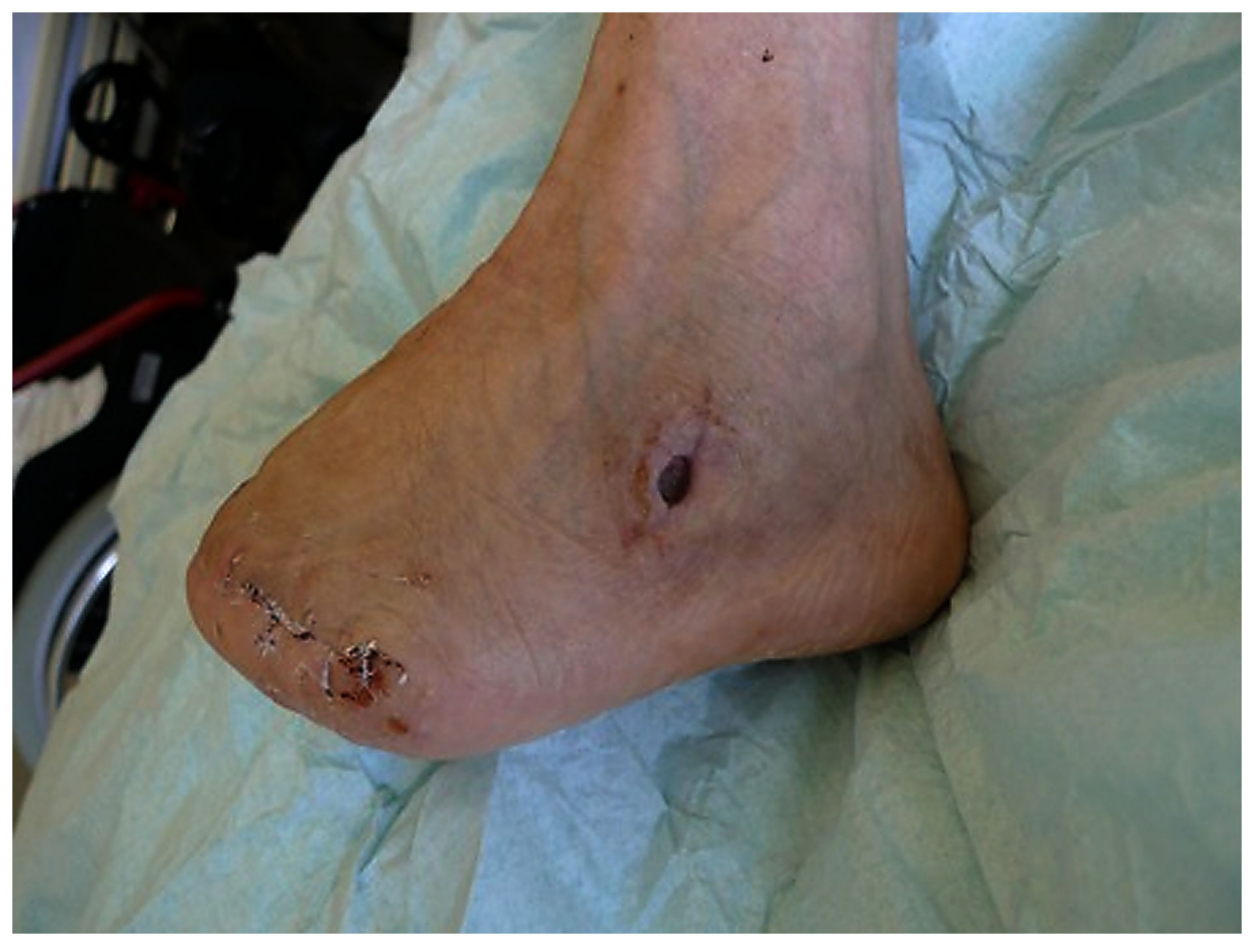
Same patient with a new defect on the lateral foot after reconstruction of the medial foot with a parascapular flap. The new defect leaves a defect of bone, that requires intensive surgical debridement and coverage.

**Figure 8:**
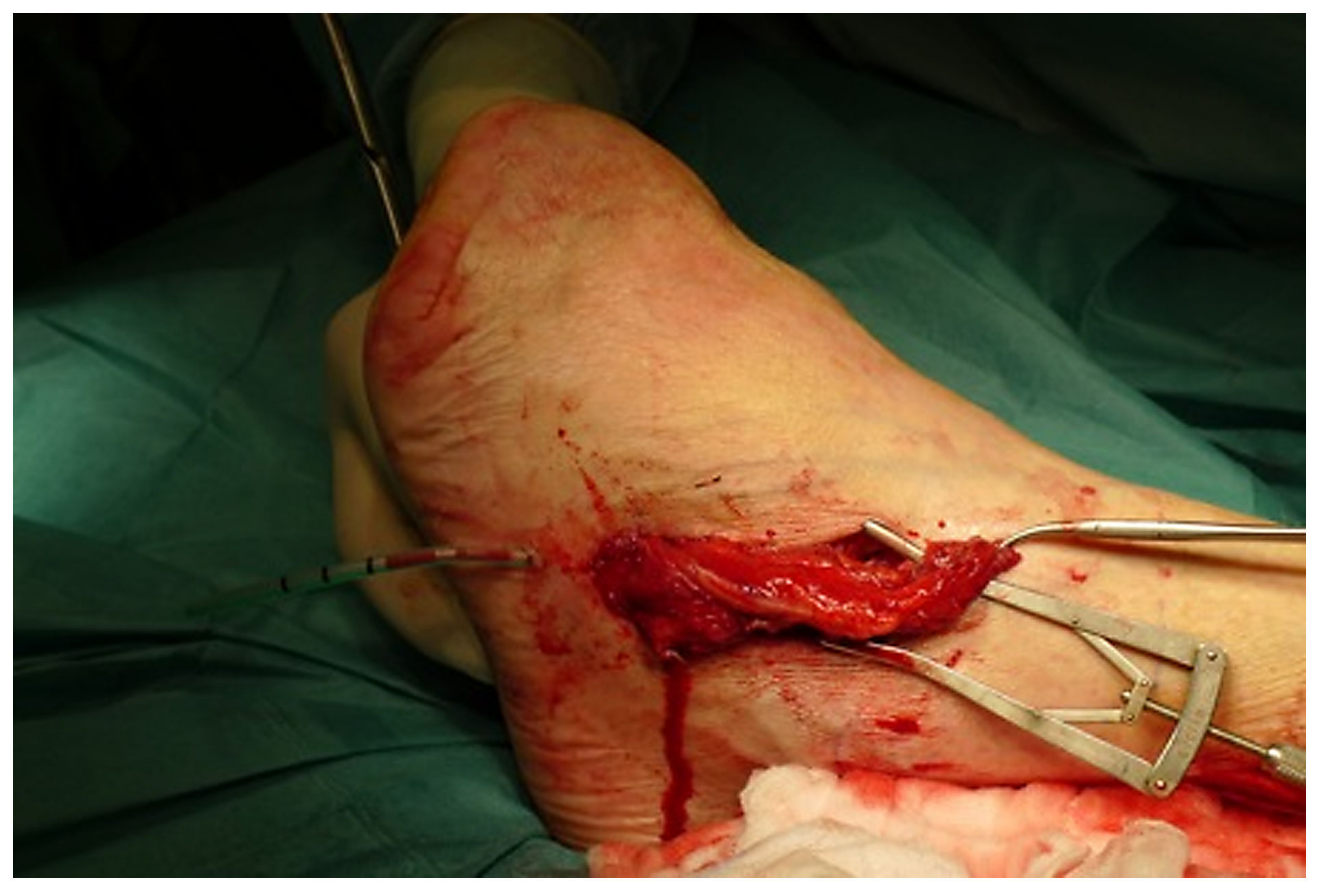
Intraoperative situs: the free peroneus brevis muscle flap, obliterates and closes the defect (flap anastomosis to the lateral tarsal artery).

**Figure 9:**
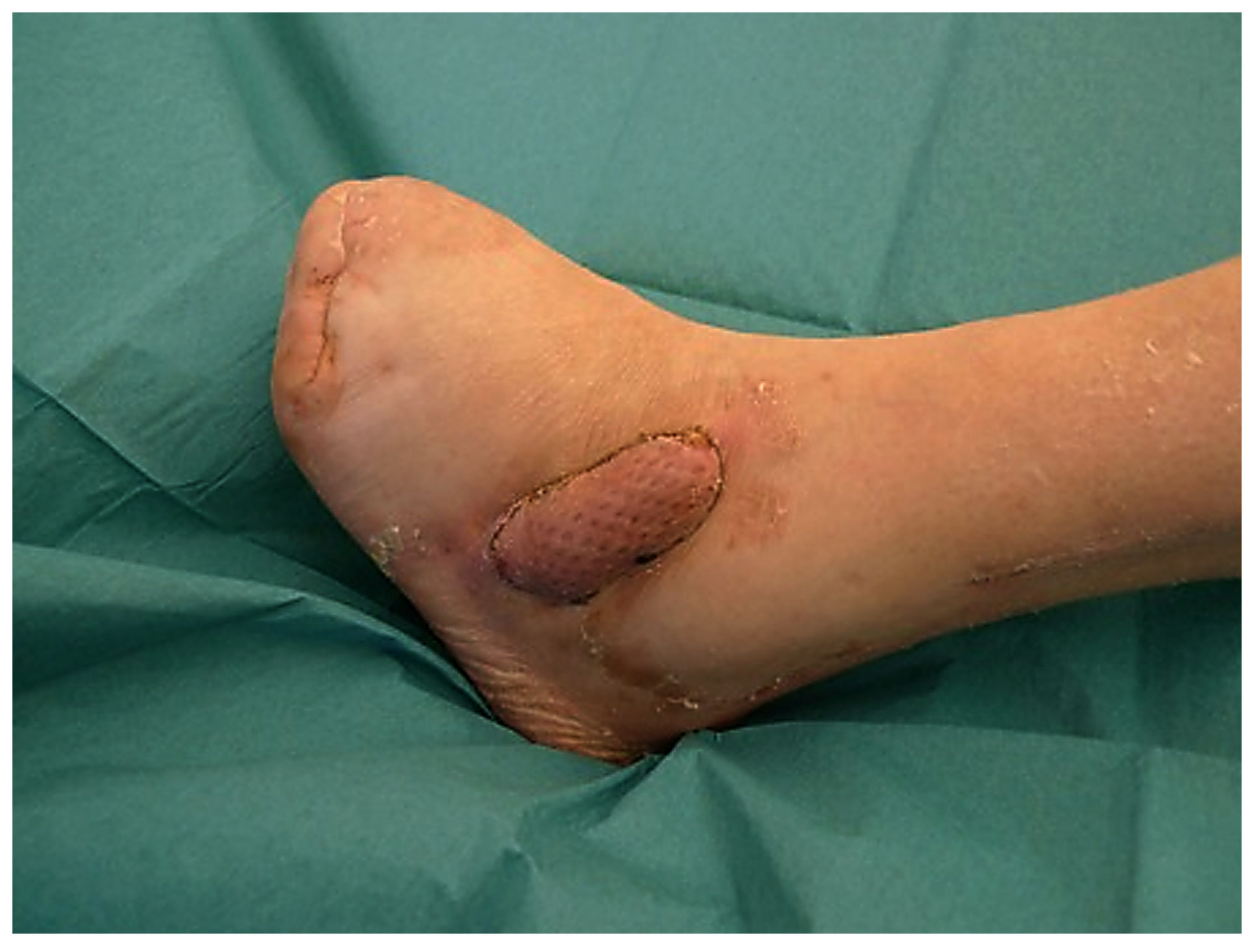
6 weeks after reconstruction. The muscle is covered with a split thickness skin graft.

The arterialized venous flap was used as a reversed popliteo-pedal bypass to the medial plantar artery, representing a bypass and flow through arterialized venous flap. 3 efferent veins were anastomosed to subcutaneous veins at the medial foot. It included a skin and subcutaneous island flap from the perforator system of the greater spahenous vein at knee level (See fig 1-5). Using a small flap for the same foot, which had already been reconstructed with a free microvascular parascapular flap is demonstrated in figures 6-9. In this case a microvascular peroneus brevis flap has been anastomosed to the lateral tarsal artery. Usually the small flaps were either anastomosed to the lateral tarsal artery, the dorsal metatarsal artery or the medial plantar artery, all representing a diameter of less than 1 mm.

Histological and microbiological specimen were taken in all cases. In all cases an extensive surgical débridement was performed prior to reconstruction. When osteomyelitis was diagnosed, the duration of antibiotic therapy was set for 6 weeks [20,21]. Patients were treated with anti platelet therapy and low molecular heparin postoperatively.

## Results

There were 2 flap losses due to a late venous thrombosis leading to amputation. Despite salvage attempts both flaps could not be rescued. This occurred in one parascapular flap and one peroneus brevis muscle flap. Furthermore, 4 Patients required an amputation within 6 months after reconstruction: 2 of them suffered from a bypass failure and ischemia of the lower extremity. 2 other patients developed a foudroyant infection of the foot requiring resection of major parts of the foot skeleton, resulting in an instable situation, and thus amputation was performed. Both had a servere phlegmonous infection prior to reconstruction, one of them was a patient with end-stage renal disease with hemodialysis. In total there were 6 amputations, which needed to be performed after the microvascular reconstruction.

Another 4 patients died within 1 year after reconstruction. 3 died of heart failure and stroke in a rehabilitation clinic, one patient died due to a cerebral haemorrhage 1 year after reconstruction. Amputation and perioperative mortality were regarded as major complications and are illustrated in table 2.

**Table 2:** Demonstrating major complications in the population of 41 diabetic patients (44 flaps).

Major complications	Amputation due to bypass failure	Amputation due to thrombosis of the flap vessels	Amputation due to foudroyant infection	Perioperative mortality
Number of patients	2	2	2	0

As hemodialysis is discussed to have a rather negative influence regarding reconstructive outcome, the results of the 3 patients, who needed hemodialysis because of an end-stage renal disease are noted as followed: 1 patient had to be amputated; 1 patient died a year after reconstruction (see above). The other patient had an uneventful healing.

There were 5 patients with a prolonged healing period due to hematoma or minor superficial necrosis of the flap. After a débridement further healing went uneventfully. Ambulation of the other patients began 10 days postoperative. Only one patient, who had an above knee amputation of the contralateral side, needed a longer period of time until ambulation.

5 patients required a second admission within 6 months postoperative. They developed new ulcers on the ipsilateral foot. 2 of them needed a new microvascular flap. 3 of them had ulcers on the flap surface due to insufficient footwear. After surgical débridement local wound therapy could be successfully performed.

## Discussion

Preventing and treating foot ulcers in diabetic patients is a very important task. A lot of strategies and innovations have been published. They include therapy of neuropathy and peripheral vascular disease, which are the leading causes for foot ulcers in diabetic patients. Decompression of nerves is a successful tool in treating neuropathy of the foot and [3,22,23,24]. Vascular disease can be treated by revascularization of the extremity, which is supposed to support healing of chronic wounds [25]. However, the first step must be a radical debridement. Having in mind that the vascular situation can be improved by bypass reconstruction and free flap coverage is possible, the debridement can be as radical as necessary.

The combined procedure of bypass reconstruction and free flap transfer is rarely performed, although it improves the perfusion of the foot and closes the defects in a single stage operation. In addition, the flap hooked on the bypass increases the flow through by reducing distal resistance [12,26,27,28,29]. Long- time results have demonstrated a rate of over 50% of Limb salvage after combined bypass- surgery and free flap reconstruction. These results are of importance, because vascular reconstruction of an ischemic limb alone is not always leading to healing of an ulcer with exposed bones or other functional structures. Also a postoperative infection of the bypass can occur, if the defect is still present. The study by Illig et al demonstrated a limb salvage of 57% and an overall survival of patients of 60% after 5 years [29]. 65% of their patients, who might have been amputated, remained in an ambulatory state. The author concludes that a primary single staged surgery might be a better choice for a combined surgery.

It is a common problem that the graft patency cannot be demonstrated very well. Illig et all have also commented on that [29]. We were able to demonstrate this phenomenon in one patient of our study who developed a contralateral ulcer 2 years after reconstruction, and thus required angiography. The reconstructed poplito-pedal bypass with the hooked parascapular flap was also visualized. Bypass and flap anastomosis were patent; and the perfusion of the foot was increased [12].

Usually patients who suffer from diabetic foot syndrome in Wagner/Armstrong stadium 3D and 4D have persistent chronic wounds or still receive amputations. However, we were able to demonstrate that amputation can be also prevented in these stages. Treatment requires an interdisciplinary approach of endocrinologists, vascular surgeons and plastic surgeons [30,31]. If necessary, free tissue transfer has to be performed. Several studies show that this can also be done successfully in older diabetic patients [11,12,32]. These patients, however, suffer from severe co- morbidities. Their entire vascular system is insufficient due to the hyperglycaemic condition. Many of them have coronary disease and chronic renal insufficiency [29]. Free tissue transfer in patients with end stage renal disease and diabetes was considered as a procedure with adverse outcome [29,33]. But nephropathy is also responsible for prolonged wound healing [34,35]. This is one of the reasons why surgical procedures may become necessary. The results in our group demonstrate that patients treated with hemodialysis for end stage renal disease must not necessarily experience flap loss. They do, however, need to be monitored carefully for bleeding and postoperative infection. Postoperative amputation, occurring in one of the patients was due to a foudroyant infection 5 days postoperatively. The flap was vital. In addition there are recent studies, which encourage limb salvage in these patients [36].

Pushing microsurgical frontiers to supramicrosurgery can help to reduce donor site morbidity. Anastomosing those small vessels requires high microvascular skills. Majority of publications describe free microvascular muscle flaps or fascio-cutaneous flaps for reconstruction [12,37,38,39]. The 6 flaps in our study were used in smaller defects. These flaps were free peroneus brevis muscle flaps, extensor digitorum brevis muscle flap, and a free arterialized venous flap from the thigh. The peroneus brevis muscle flap is usually applied as distally pedicled flap. It can be used for defects around the ankle. However, the distal pedicle limits perfusion and the rotation arc. We therefore used the flap as free flap to close distant defects at the foot. Although, this flap is not a standard treatment option in smaller defects it may represent a valuable option. Although atherosclerosis is present in major vessels, smaller perforator vessels seem to be less affected. This has also been described by Hong [14]. The harvest of these small flaps is fast and less traumatic than harvesting a larger muscle or fascio-cutaneous flap. Donor site morbidity is limited. Although fascio-cutaneous flaps are supposed to have lower donor site morbidity than muscle flaps, there is evidence indicating decreased muscle strength after harvesting anterior lateral thigh flaps [40]. Koshima described supramicrosurgical flaps from the lower abdomen as true perforator flaps (DIEP-flap) [13]. The peroneus muscle flap is not a true perforator flap but offers a constant pedicle and muscle belly not requiring a thinning procedure as fascio-cutaneous flaps.

The arterialized venous flap was used as a popliteo-pedal bypass with a skin and subcutaneous island flap from the greater spahenous vein. The vein itself was used as bypass to revascularize the foot. By harvesting cutaneous tissue around the vein a flap was created which resembles a so called venous flap. As the vein graft was used to bridge the artery the cutaneous tissue becomes arterially vascularized. These flaps however require a venous outflow. We therefore used a couple of subcutaneous efferent veins to establish venous drainage. This flap type can also be used in traumatic defects of the lower leg and foot. It is able to cover a defect and to increase the perfusion of the foot in one manoeuvre.

Using the extensor digitorum brevis muscle flap spared donor site morbidity. It limited the surgical area to the foot. The nutrient vessels were spared from arteriosclerosis and could be anastomosed to the dorsal metatarsal artery in supramicrosurgical fashion.

Santanelli et al [41] described the sensory reconstruction of the sole of the foot. However, only one patient in the population suffered from diabetes. They conclude that in young patients nerve coaptation can improve stability of the flap and should be considered. Patients in this study were not treated in such manner. The authors consider the perceived advantage of this procedure for patients with neuropathy inconclusive.

Major amputation represents a loss of life quality. Additionally, mortality rises after amputation. Patients often experience fundamental changes in their social lives. Many need to move into a retirement home, nursing facility or are even hospitalized due to morbidities caused by immobility [42]. Microsurgical reconstruction of the foot has proven to provide a fast psycho-physical and social rehabilitation [43]. Arguments regarding good prosthetic care will enable mobility may be correct in young patients. Elderly diabetic patients have difficulties putting on a prosthesis without support. In many cases their neuropathy has already limited their hand and finger coordination. In addition they suffer from diabetic retinopathy.

Before indicating limb salvage by free tissue transfer, the patient has to be consulted and included into decision making. The technique, possible complications and the follow up have to be explained very precisely. Moreover the patient needs to be compliant.

## Conclusion

Free tissue transfer is a well established and safe procedure for coverage of defects after trauma and cancer. Diabetic patients with a diabetic foot syndrome often need surgical treatment for wound coverage. In addition to a radical débridement and vascular surgery, free tissue transfer may also be necessary in order to prevent major amputation. Besides well established free flaps, it is possible to use smaller flaps with smaller vessels for coverage. They help reducing donor site morbidity and spare major vessels of the foot. Limiting factors of successful treatment are co-morbidities such as cardiovascular and renal disease. Nevertheless, every attempt should be taken to prevent major amputations in diabetic patients. Interdisciplinary treatment enables us to achieve this goal. The results in this publication are very encouraging for other diabetic patients.
